# Okadaic Acid Depuration from the Cockle *Cerastoderma edule*

**DOI:** 10.3390/toxins14030216

**Published:** 2022-03-16

**Authors:** Juan Blanco, Helena Martín, Carmen Mariño, Araceli E. Rossignoli

**Affiliations:** Centro de Investigacions Mariñas (CIMA), Xunta de Galicia, Pedras de Coron s/n, Vilanova de Arousa, 36620 Pontevedra, Spain; helena.martin.sanchez@xunta.gal (H.M.); maria.carmen.marino.cadarso@xunta.gal (C.M.); araceli.escudeiro.rossignoli@xunta.gal (A.E.R.)

**Keywords:** kinetics, Michaelis-Menten, models, esterification

## Abstract

The cockle *Cerastoderma edule* is a commercially important species in many European Countries. It can accumulate okadaic acid (OA) and other toxins in its group, which makes it unsuitable for human consumption, producing harvesting bans to avoid intoxications. The duration of those bans depends in part on the depuration kinetics of the toxin in this species. In this work, this kinetics was studied by means of fitting different models to depuration data experimentally obtained, using naturally contaminated cockles. Cockles depurated OA faster than most other bivalve species studied. Models that include Michaelis-Menten kinetics describe the depuration better than those using a first order exponential decrease to describe the first (or the only) compartment. One-compartment models were not able to describe the final part of the depuration curve, in which OA was depurated very slowly. Therefore, two-compartment models were needed. Esters were depurated at a much faster rate than the free form of the toxin; however, no significant esterification was detected during the process. The slow depuration rate suggests that other bivalve species could be used as sentinels to monitor cockle populations, but caution should be taken when toxin concentrations are very high.

## 1. Introduction

Okadaic acid (OA) and dinophysistoxins (DTX) are compounds produced by several species of marine dinoflagellates and accumulated by other organisms, mainly bivalves. These toxins, when consumed by humans and other mammals, produce a syndrome known as diarrhetic shellfish poisoning (DSP). In benthic environments, several *Prorocentrum* species can produce these toxins [1,2,3,4]. In plankton, the main producers are species of *Dinophysis*, which can contain OA, DTX1, DTX2, some isomers, some derivatives, such as diol- and triol-esters [5,6,7,8,9], and occasionally the groups of compounds known as DTX4 and DTX5 (up to now not described in *Dinophysis* but found in some OA-producing benthic species) [10,11]. *Dinophysis* species are distributed worldwide and DSP intoxications by mollusk consumption have been reported from many countries [6]. After the discovery of DSP by Yasumoto et al. [12] and the development of an assay to quantify it, the detection of this toxicity was progressively incorporated into shellfish monitoring systems. Later, the development of analytical methods to quantify these toxins allowed for the creation of reference levels for their maximum content in bivalve soft tissues, which cannot be surpassed to consider the shellfish safe for consumption. Currently, in the European Union [13] and most other countries, this level has been established at 160 µg of OA-equivalents kg^−1^, a level which was established from an acute reference dose of 0.3 µg of OA kg^−1^ of body weight obtained from epidemiological data [14]. In many areas, the recorded toxin concentrations led to frequent bans of mollusk harvesting, which represent an important economic and social problem.

Mollusk, and especially bivalves, are appreciated as a source of food for humans and, consequently, their fisheries are commercially important. The cockle *Cerastoderma edule*, among the non-cultured mollusks, is one of the economically most important species in Europe. Its production between 2014 and 2017 ranged from 14,651 to 26,125 *t*, with the UK as the top producer and Spain the second [15]. In Galicia (NW Spain) the annual production is around 600 *t* [16], and represents an important resource for the people who gather shellfish from the intertidal zone (a regulated activity). Bans can produce significant economic losses in the productive sector, which are dependent on their duration.

Ban duration depends on the amount of toxins that the bivalves can ingest, which in turn depends on the abundance of toxic cells, the toxin contents of the cells, the time during which the toxic populations persist, and the depuration kinetics of the bivalve [17]. Banning periods could be substantially shortened for the species that depurates faster. For *C. edule*, OA depuration rate has only been roughly estimated but seems to be faster than that of the mussels *M. galloprovincialis* and *M. edulis* [18,19], and some clams and oysters (reviewed in [17]). Its depuration kinetics has not been studied, making it difficult to precisely predict the time course of its reduction of toxicity.

In this work, the depuration kinetics of OA (free and esterified form) in *C. edule* has been determined by means of the implementation and fitting of different models to the data obtained using cockles naturally contaminated with the toxin.

## 2. Results

Okadaic acid was present in cockles mainly esterified with fatty acids. Initially, only 1.8% of the toxin was in free form, but attained 18.7% after 19 days of depuration, decreasing thereafter to 2.9% on day 33. In the experiment, free OA constituted 8.1% of the total toxin, on average. There was not a lineal trend of the percentage of free OA during the course of the experiment, nor was there any relationship with the change in body weight.

**Figure 1:**
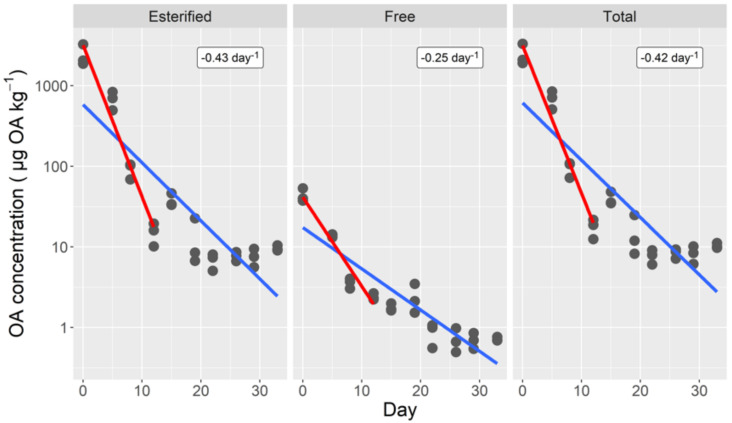
Depuration curves of esterified, free, and total okadaic acid of the cockle. The lines correspond to fitting a simple exponential decrease to all data (blue line), and to the first 12 depuration days (red line). The numbers in the upper right corners are the estimated depuration rates for the first 12 days.

Both esterified and total toxin decreased quickly during the first 12 days of depuration—they were, at day 5, below 30% of the initial level and below 1% at day 12—but the decreasing velocity slowed down during subsequent days. The trend was similar for free toxin, but the transition was softer and the depuration velocity smaller (Figure 1).

**Figure 2:**
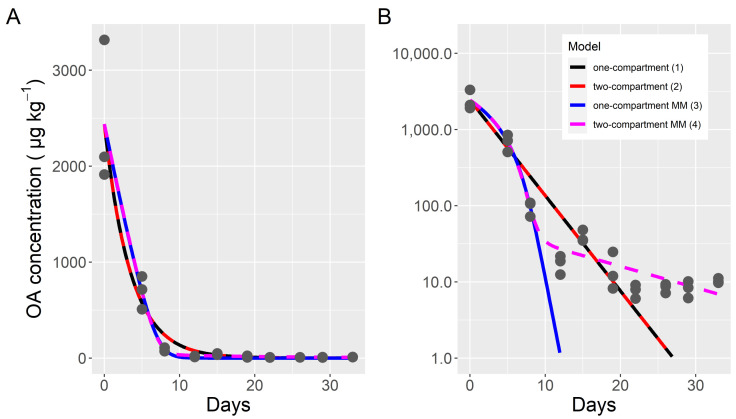
Fitting of four models to the total okadaic acid depuration data of the cockle, in untransformed (**A**) and logarithmically transformed (**B**) scale. Dots are the observed data and lines are the outputs of the fitted models. MM indicates that the model used Michaelis-Menten kinetics for the first compartment (models 3 and 4).

The depuration kinetics of the total toxin, clearly, did not follow an exponential decay. A one-compartment model does not fit the obtained data correctly. It overestimates the middle part of the curve and underestimates the final part, as can be observed from the depart of linearity of the logarithmically transformed data (Figure 1). All attempts to fit a two-compartment model with exponential kinetics were unsuccessful, because the fitted parameters, in fact, reverted it to a one-compartment model (Figure 2). By assuming that the depuration followed Michaelis-Menten kinetics, the model fit the initial and middle part of the curve much better, but the toxin content of the final part, when the toxin content was very low, was substantially underestimated, even when the differences were quantitatively small (only noticed in the logarithmically transformed data (Figure 2). A final model that combines Michaelis-Menten kinetics for the first compartment and exponential kinetics for both the transfer between compartments and the depuration from compartment 2, fit the data much better, even in the final part of the curve (Figure 2).

**Figure 3:**
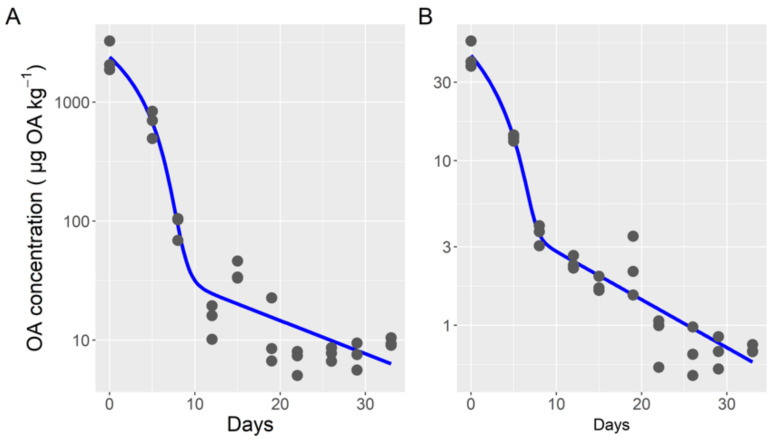
Depuration curve (dots) and output of the two-compartment model (model 4) with Michaelis-Menten kinetics for the first compartment (lines), for esterified (**A**) and free (**B**) okadaic acid.

The two-compartment Michaelis-Menten model also fit the data for esterified and free OA well (Figure 3). The fitted parameters, however, were not the same for the two forms. As could be expected by its high contribution to the total toxin, the parameters obtained for esterified okadaic acid were very similar to those for total toxin, but both Vmax and Km were slightly higher. Contrarily, some of the parameters obtained for free OA were very different. Those corresponding to the first compartment (Michaelis-Menten kinetics) were substantially lower, with Vmax and Km being only 1.6% and 1.2% of the corresponding rates for esterified OA, respectively. The estimated transfer rate between compartments 1 and 2 was six times higher for free than for esterified OA. The depuration rates of the second compartment were practically the same for the two OA forms.

**Figure 4:**
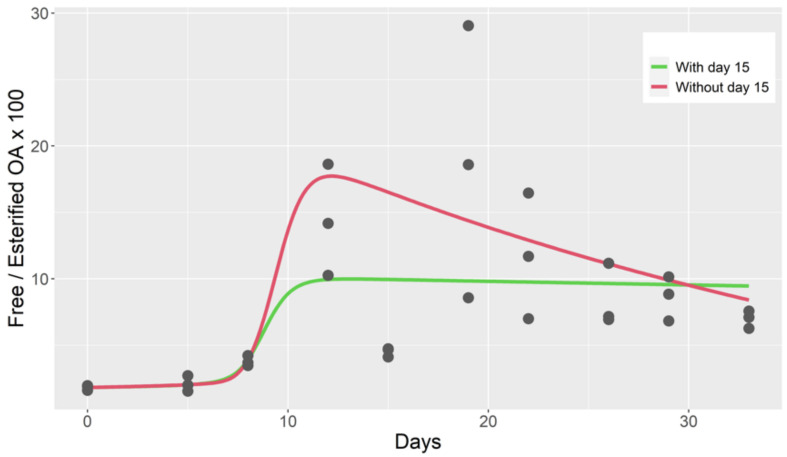
Observed free/esterified OA ratio during the depuration of cockle (dots), computed from the output of a two-compartment model with Michaelis-Menten kinetics for the first compartment (model 4).

This model also fits the ratio of free/total toxin reasonably well when the data of day 15, which were atypically low, were removed (Figure 4). When an esterification rate was included in the model (model 5), the fit to the data did not improve.

In order to be able to compare the observed depuration rates with other species, an exponential decay (model 1) was assumed for the first 12 days (in most cases in the available literature, depuration rates are computed assuming a first order exponential decay, or the data obtained are not enough to re-compute them to fit a more complex model). In such a case, rates of 0.42, 0.43, and 0.26 day^−1^ were obtained for the total, esterified, and free toxin, respectively (Figure 1), which correspond to 1.7, 1.6, and 2.7 days of semi-depuration period (time required to reduce the toxin concentration by 1/2).

## 3. Discussion

Cockles depurate most of their OA content faster than other studied bivalve species. They can reduce their toxin content by a half in 1.7 days. The rates observed in the mussels *M. galloprovincialis* and *M. edulis* are substantially lower than those found in this study, ranging from 0.05 to 0.19 day^−1^ [19,20,21,22,23,24,25,26] (reviewed in Blanco [17]). This is also the case with different infaunal bivalve species such as *Spisula solida* [27] and *Donax trunculus* [26,27]. Vale et al. [19] also found higher depuration rates of OA and DTX2 in cockles (0.22 day^−1^, recomputed from the fastest decay of the depuration curve) than in mussels (0.09 day^−1^).

The better fitting of the models, which include the Michaelis-Menten kinetics in relation to those using an exponential decay, suggests that a saturable transporter should be involved in the process. Consequently, when the amount of toxin accumulated is very high, the depuration is slower, in relation to the toxin concentration, than when the concentration is lower. This kind of response seems to also be present in the depuration data obtained by Vale et al. [19], but it is not frequent among bivalves, whose depuration usually follows an exponential decay [17]. The need of a second compartment, with very low depuration and transfer rates, in the models of total toxin indicates that there is a small amount of nearly residual toxin that could persist in cockles for a long time. The amount of this nearly residual toxin is very low and does not pose any risk for consumer health. The presence of a small second compartment for OA is frequent among bivalves [17].

Most toxin was found to be in esterified form. Esterification of xenobiotics with fatty acids seems to be a frequent step in depuration, and has been shown to take place with okadaic acid and analogs [28] and with steroids [29]. Most bivalves quickly esterify okadaic acid, making esters constitute more that 90% of the total toxin [17].

Esters are depurated from the cockle at a much higher rate than free toxin. This difference was also found in a previous study with the same species in Portugal [19], and was also the case in the mussels *M. galloprovincialis* [19] and *M. edulis*, but not in the oyster *Ostrea edulis* in Norway [30]. However, Lindegarth et al. [31], also from Norway, reported that free forms were depurated from mussels and oysters faster than the esters. Notwithstanding, an approximate re-computation from their plots using only the first two depuration weeks shows that the esterified forms depurate at a much higher rate (nearly 2x) than the free toxin in both species. The fact that the fit to the data did not improve when esterification of the free OA was included in the model suggests that this process had low importance during cockle depuration. This is surprising because it is known that free OA is easily esterified in bivalves [28,32,33] and, during four years of monitoring in Galicia, the percentage of free OA in cockle was nearly always below 5% of the total toxin [34], which would not be possible if the esterification was not very fast. One explanation for this is that most OA was already esterified at the beginning of the experiment (more than 98% of the OA), making it difficult to estimate the model parameters precisely. Another possible explanation would be that the esterification rate follows a sigmoid curve, with low esterification rates when the concentration of free OA is also low (due to inhibition by the product or other causes). Rossignoli et al. found that most OA was depurated from the mussel *M. galloprovincialis* in esterified form [35], even when the proportion of free form in that species is high, which suggests that free OA should be esterified before being excreted from the digestive gland cells. This would explain why the second compartment is relatively more important for free that for esterified OA.

The fast depuration rate observed suggests that cockles represent a smaller risk than other bivalves and, consequently, that other less valued species, such as mussels, can be used as sentinels to carry out an efficient monitoring system. However, some precaution should be taken when very high toxin concentrations are attained because, in those cases, with the observed kinetics, cockles can depurate the toxin slower than mussels and other species whose depuration follows a first-order exponential decay.

The identification of the membrane transporter most likely involved could allow for selecting fast depurating cockles or for the creation of design-specific treatments to accelerate depuration in the future.

## 4. Materials and Methods

### 4.1. Biological Material and Experimental Design

Cockles *C. edule* were obtained from the Baldaio, A Coruña, Spain (43°17′ N, 8°39′ W), where they had accumulated OA from a bloom of *Dinophysis acuminata*. They (120 individuals) were placed in a 50 L tank with running seawater at a temperature of approximately 18 °C and maintained there until the end of the experiment (33 days). A mixture of *Tetraselmis*, *Isochrysis*, *Pavlova*, and *Chaetoceros* was supplied daily, and the flow was stopped for two hours. At days 0, 5, 8, 12, 15, 19, 22, 26, 29, and 33, three samples of three cockles each were obtained randomly. The shells were removed, and the soft tissues of each 3-cockle sample were weighed and subjected to toxin extraction.

### 4.2. Chemicals and Reference Materials

Acetonitrile (LC-MS grade) and methanol (HPLC grade quality) were purchased from Scharlab (Sentmenat, Spain) and VWR (Llinars del Vallés, Spain), respectively. Ultrapure water was obtained from a Milli-Q gradient system fed with an Elix Advantage-10 (Millipore Ibérica, Spain). Ammonium hydroxide (NH_4_OH, 25%) and sodium hydroxide (NaOH > 99%) were obtained from Merck (Barcelona, Spain), and hydrochloric acid (HCl, 37%) from Panreac (Barcelona, Spain).

The OA certified solution was acquired from the Institute for Marine Biosciences, National Research Council (NRC), Halifax, Nova Scotia, Canada.

### 4.3. Toxin Extraction and Hydrolysis

Toxins from cockle tissues were extracted in methanol MeOH (1:4 *w:v*) with an Ultraturrax T25 (IKA, Staufen, Germany) homogenizer. Solids were removed by centrifugation at 18,000× *g*, and the supernatant was kept at −20 °C until analysis. Before the analysis, the possible precipitates were removed by filtration through 0.22 µm polyethersulphone PES syringe filters (Membrane Solutions,, from Jasco Analítica, Madrid, Spain).

To determine free OA, the extracts were analyzed without additional processing. To quantify total OA (free + esterified), aliquots of the extracts were subjected to an alkaline hydrolysis, following the procedure established in the SOP of the European Community Reference Laboratory of Marine Biotoxins [36], which consist of adding NaOH 2.5 M, heat at 76 °C for 40 min, and neutralizing the solution with HCl 2.5 M.

### 4.4. LC-MS/MS Method

The analyses have been carried out on an Exion LC AD™ System (SCIEX, Framingham, MA, USA) coupled to a Qtrap 6500+ mass spectrometer (SCIEX) through an IonDrive Turbo V interface in electrospray mode. The chromatographic separation [37] was made in a Phenomenex (Alcobendas, Spain) Kinetex EVO C18 column 50 mm × 2.1 mm, 2.6 µm using a gradient of water (phase A) and acetonitrile MeCN 90% (phase B), both containing 6.7 mM NH_4_OH (pH 11) [38]. The gradient started with 22% B, (held for 0.1 min), followed by a linear increment to reach 95% B at minute 1.8, and holding this composition until minute 2.90. The proportion was then returned to 22% B in 0.20 min and held for 0.5 min before the next injection. The flow rate, the injection volume, and the column temperature were 1000 µL min^−1^, 1 µL, and 40 °C, respectively.

The mass spectrometer parameters were optimized by direct infusion using toxin standards, when available, and were set to: Ion source Gas 1, 75 (arbitrary units); Ion source Gas 2, 75 (arbitrary units); and −4500 V. The transitions 803.5 > 255.1 and 803.5 > 563.4, with collision energies of −62 and −60 v, were used as quantifier and qualifier, respectively.

The OA in the extracts was quantified by the external standard method using dilutions of a certified solution of OA in MeOH.

### 4.5. Modelling

Models were implemented using the R [39] package deSolve [40] and fitted to the observed data with the package FME [41]. The results were plotted with the R package ggplot2 [42].

Five models were fitted. Model 1 was a simple exponential decay:

dOA/dt = −K·OA(1)

where K is the depuration rate.

Model 2 also used the simple exponential decay of the toxin but from two compartments, depurating the first one faster than the second, and transferring a part of its toxin content to the second compartment.

dOA_1_/dt = −K_1_·OA_1_ − T_12_·OA_1_ (2)

dOA_2_/dt = −K_2_·OA_1_ + T_12_ (3)

OA_T_ = OA_1_ + OA_2_ (4)

where subscripts indicate the compartment and T_12_ is the transfer rate from compartment 1 to 2.

The third and fourth models are analogs of the first and second, respectively, but the rate of the exponential decay in the unique (model 3) or the first (model 4) compartment was replaced by the Michaelis-Menten parameters.

Model 3:

dOA/dt = −Vmax·OA/(Km + OA)(5)

where Vmax is the maximum depuration velocity and Km is the Michaelis-Menten constant.

Model 4:

dOA_1_/dt =−Vmax·OA_1_/(Km + OA_1_) − T_12_·OA_1_ (6)

dOA_2_/dt = −K_2_·OA_1_ + T_12_·OA_1_ (7)

OA_T_ = OA_1_ + OA_2_ (8)

Model 5:

dOAest_1_/dt = −Vmax_est_·OAest_1_/(Km_est_ + OAest_1_) − Test_12_·OAest_1_ + Kest·OAfree_1_ (9)

dOAest_2_/dt = -Kest_2_·OA_1_ + Test_12_·OAest_1_ (10)

dOAfree_1_/dt =−Kfree_1_·OAfree_2_ − Tfree_12_·OAfree_1_ − Kest·OAfree_1_ (11)

dOAfree_2_/dt = −Kfree_2_·OAfree_2_ + Tfree_12_·OAfree_1_ (12)

OAest_T_ = OAest_1_ + OAest_2_ (13)

OAfree_T_ = OAfree_1_ + OAfree_2_ (14)

where “free” or “est” indicate if the toxin is in free or esterified form, and Kest is the rate of esterification of the free toxin (only in compartment 1).

## Data Availability

All the data used in this study are available from the article.
